# GDF15 affects venous thrombosis by promoting EndMT through smad2/p-smad2 pathway

**DOI:** 10.1186/s12959-023-00547-7

**Published:** 2023-09-18

**Authors:** Yeping Li, Huiqi Zhu, Wanghao Xin, Jiaoyan Wang, Chao Yan, Kejing Ying

**Affiliations:** Department of Respiratory Medicine, Sir Run Run Shaw Hospital, School of Medicine, Zhejiang University, 3 Qingchun East Road, Hangzhou, Zhejiang 310016 China

**Keywords:** Venous thromboembolism, Growth Differentiation Factor 15 (GDF15), Endothelial-to-mesenchymal transition (EndMT)

## Abstract

**Background:**

Endothelial-to-mesenchymal transition (EndMT) is a pathophysiological change in the vascular endothelium commonly seen in the cardiovascular system. Elevated serum Growth differiention factor 15 (GDF15) has been reported in VTE patients, but the relationship and mechanism between GDF15, EndMT and VTE are still unclear.

**Methods:**

We performed a retrospective clinical study, and human serum GDF15 expression levels were detected. The mouse DVT model was established through subtotal ligation of the mouse inferior vena cava, and then we detected intimal changes and thrombi in the stenotic inferior vena cava by haematoxylin-eosin (HE) staining, Masson staining, and Sirius Red staining. The expression levels of GDF15 and SM22 were detected by immunohistochemistry and RT‒qPCR. Serum samples of mice were collected, and the expression level of GDF15 in serum was detected. Human umbilical vein endothelial cells (HUVECs) were stimulated with a cytokine mixture (TGF-β1 + TNF-α + IL-1β). The role and mechanism of GDF15 in EndMT and VTE were detected in HUVECs and in a DVT mice model.

**Results:**

We found that serum GDF15 levels in both VTE patients and mouse DVT models were higher than those in the control group. EndMT was increased in the stenotic vascular tissue of mice. Further experiments showed that GDF15 could promote the EndMT of HUVECs and reduce their anticoagulation and antifibrinolytic ability through the smad2/p-smad2/snail pathway. Inhibition of mature GDF15 release can significantly reduce venous thrombotic fibre deposition in mice.

**Conclusions:**

GDF15 positively promotes EndMT through activation of the Smad2/psmad2/snail pathway, and inhibition of GDF15 expression can alleviate the EndMT process, further improving the coagulation and fibrinolytic function of endothelial cells and thus reducing the local fibre deposition of venous thrombi.

**Supplementary Information:**

The online version contains supplementary material available at 10.1186/s12959-023-00547-7.

## Introduction

Venous thromboembolism (VTE) is a serious condition that includes both deep vein thrombosis (DVT) and pulmonary embolism (PTE). VTE is responsible for approximately 10% of all inpatient deaths [1]. Both PTE and DVT share the same risk factors and are different clinical presentations of VTE in various parts and stages of the body. DVT most commonly occurs in the leg veins but can also occur in the visceral, cerebral, and arm veins [2]. VTE affects nearly 10 million people worldwide every year and is an important factor in the global burden of disease [3]. Although the death rate within 30 days of the onset of PTE has been declining in recent years with advances in medical technology, 20% of people with PTE still die before or shortly after diagnosis. VTE is also a chronic disease, and approximately 30% of VTE patients will relapse within 10 years [4]. In recent years, the number of diagnostic cases of VTE has increased rapidly. As shown by domestic statistics, the proportion of PTE among inpatients increased from 0.026% to 1998 to 1.45% in 2008 [5].

Since December 2019, the coronavirus disease-19 (COVID-19) pandemic has led to some thrombus-related problems in patients infected with COVID-19, such as the formation of large and microvascular thrombosis due to an increased risk of high blood clotting [6]. VTE is a major cause of death in hospitalized patients with COVID-19, and the incidence of VTE in intensive care unit (ICU) patients can reach 1/3, even in patients receiving preventive anticoagulant therapy [7]. Meta-analysis has shown that the prevalence of VTE, DVT, and PTE in hospitalized patients with COVID-19 is approximately 19-59.8%, 18.1-69.2%, and 13.5-27.8%, respectively [8]. The incidence of VTE in the ward or intensive care unit of COVID-19 patients was 7.1% and 27.9%, respectively [9]. In autopsy cases, the incidence of VTE is as high as 60% [10, 11].

EndMT is a complex biological process in which endothelial cells lose endothelial characteristics, acquire mesenchymal phenotypes, and express mesenchymal cell markers such as α-SMA and Fsp1. EndMT is induced by inflammation and leads to tissue fibrosis, pulmonary hypertension, atherosclerosis, and other pathological states through vascular system dysfunction [12]. Current evidence suggests that inflammation-induced EndMT is similar to EMT and is mainly controlled by two signalling pathways: the TGF-β pathway and the non-TGF-β pathway [13]. Although inflammatory-mediated signalling pathways have been extensively studied [14], the cellular signalling associated with inflammatory-induced EndMT remains poorly understood.

GDF15 was first isolated from the human bone marrow mononuclear cell line U937 in 1997 and identified as macrophage inhibitory cytokine 1 [15], also known as Prostatogenic factor, placental transforming growth factor-B, placental bone morphogenetic protein, nonsteroidal anti-inflammatory drug activating Gene-1, etc. It is a member of the TGF-β cytokine superfamily of the stress response. The sequence conservation rate with other superfamily members was 15–29% [16]. GDF15 is considered to be a marker of oxidative stress, inflammation, and tissue remodelling [17–21]. In recent years, GDF15 has been found to be involved in the occurrence and development of a variety of diseases, including cardiovascular diseases, kidney diseases, liver diseases, metabolic syndrome, diabetes, and septicemia [16]. It has been shown to be a biomarker of future cardiovascular disease risk and risk of thromboembolism and bleeding in patients with atrial fibrillation treated with anticoagulants [22, 23]. Serum GDF15 levels were significantly increased in hospitalized patients with pulmonary embolism [24]. There have been many clinical reports on GDF15 in VTE, mainly in foreign countries. However, there are limited studies on whether GDF15 is different in different VTE populations and the specific mechanism it plays in the occurrence and development of VTE.

It has been reported that GDF15 promotes the progression of EndMT in endothelial cells in patients with lipodystrophic syndrome [25]. In the case of VTE, the relationship between GDF15 and VTE and EndMT is unclear. This study intends to further establish animal models and cell experiments through clinical studies to explore the relationship between GDF15 and EndMT in VTE, as well as its role and possible mechanism.

## Materials and methods

### Human samples

Blood samples (matched by sex and age) were collected from hospitalized VTE patients and physical examinations were conducted from January 2023 to April 2023. The patient’s blood samples were approved by the Ethics Committee of Run Run Shaw Hospital Affiliated with Zhejiang University School of Medicine. The serum specimens were packed according to the experimental requirements and transferred to a -80℃ refrigerator for preservation.

### Mouse thrombus model

SPF grade, 8-week-old male C57 mice were reared in the Laboratory Animal Center of Run Run Shaw Hospital Affiliated with Zhejiang University. The feeding environment was kept at a constant temperature (25 ± 2) ℃ and constant humidity (50 ± 5) %. Artificial light was on 12 h each day. The animal experiments were approved by the Animal Ethics Committee of Run Run Shaw Hospital Affiliated with Zhejiang University School of Medicine. The process of establishing the mouse model of DVT was as follows. Mice were anaesthetized with 1% pentobarbital sodium (50 mg/kg), and the operation area was disinfected with iodophor. The abdomen of the mice was cut with line scissors along the midabdominal line, followed by the epidermis and muscle layer, and the abdominal cavity was exposed. The inferior vena cava was exposed after the intestinal tract was turned over, and the blood vessel was bluntly separated with sharp forceps. The main inferior vena cava below the left renal vein was carefully ligated, and the 5 − 0 suture was extracted. The mice were put back into the cage and recovered after half an hour to 1 h after successive sutures were used to close the intraperitoneal muscle layer and intermittent sutures to close the epidermis. The mice were again disinfected with iodophor. The mouse model samples were collected 1 week later. Twenty-one mice were randomly divided into the control group and the DVT group. There was no significant difference in body weight between the two groups. Decanoyl-RVKR-CMK treatment was administered by caudal vein injection (25 mg/kg) on the 1/3/5th day of modelling, and blank treatment was administered by an injection of the same amount of normal saline.

### Immunostaining

Mouse vein tissue was fixed with 4% paraformaldehyde fixation solution for 24 h and cut into 0.2 cm tissue sections. After treatment, the slices were sliced at a thickness of 4–6 μm. HE staining, Masson staining, and Sirius red staining were performed. The primary antibody (Abmart, TD6006S) and secondary antibody (CST) against GDF15 were used for immunohistochemistry. Beyotime Biotechnology (AF5318) and fluorescent secondary antibodies (CST) were used for immunofluorescence.

### HUVECs culture

HUCECs were purchased from Zhejiang Meisen Cell Technology Co., Ltd. ECM culture medium containing 5% foetal bovine serum + ECGs + 100 units/mL penicillin and 100 μg/mL streptomycin was used. Cells were stimulated with TGFβ1 (5 ng/ml) + TNFα (5 ng/ml) + IL1β (0.1 ng/ml) (Pepro Tech, USA) for 24 h after starving for 24 h.

### Western blotting

Lysis buffer (Beyotime Biotechnology, China) and protease inhibitor (1%) and phosphatase inhibitor (5%) (Beyotime Biotechnology, China) were added to extract cell proteins. The protein concentration was detected by a BCA kit (Beyotime Biotechnology, China). Approximately 20 μg protein was added to each well during glue running. After electrophoresis and membrane transfer, the primary antibody was added overnight. The next day, after washing with PBS, the corresponding secondary antibodies (sheep anti-rabbit secondary antibody CST, sheep anti-mouse secondary antibody CST) were added, incubated at room temperature for 2 h, and exposed with an enhanced chemiluminescence kit HRP (FDbio science, China). Beta-actin was used as the internal control. The primary antibodies used in this study were: β-actin ((CST, #8457, 1:1000), SM22 (Beyotime, AF5318, 1:1000), GDF15 (Abcam, ab206414,1:1000), CD31 (CST, #77,699, 1:1000), TF (Beyotime, AF2455, 1:1000), Snail (CST, #3879, 1:1000), SMAD2 (CST, #5339, 1:1000), p-SMAD2 (CST, #7348, 1:1000), Smad2/3 (CST, #8685, 1:1000).

### Cellular immunofluorescence

The cells were laid on 24-well plates, treated with drugs, removed from the medium and washed with PBS 3 times. After fixed and broken membrane treatment, the cells were blocked with 3% BSA for 1 h and incubated at 4 °C with primary antibody overnight. The cells were washed with PBS 3 times, and the secondary antibody was added and incubated for 1 h in the dark. The cells were washed with PBS 3 times, DAPI was added, and the cells were incubated for 15 min in the dark. After washing again, a fluorescence quencher was added and observed by fluorescence microscopy.

### RNA extraction and RT‒qPCR

Cell RNA or mouse tissue RNA was extracted by the RNA-quick Purification kit. (Yishan Biotech, China) RNA concentration was measured on a NanoDrop2000 instrument. The Synthesiskit cDNA synthesis kit was transcribed by HiFiScript cDNA Synthesiskit after measurement. After that, TB Green® Premix Ex Taq™ II was added for RT‒qPCR in a LightCycler480 System. After the reaction, the corresponding cycle number (CT value) was recorded, and the relative gene expression was calculated by the 2-△ △ CT method. The primers used are shown in supplemental Table 1.

### Overexpression and knockout

The siRNAs for GDF15 were synthesized by GenePharma (Shanghai, China). The sequences were as follows: siGDF15#1 (sense: 5′-CCAAACAGCUGUAUUUAUATT-3′, antisense: 5′- UAUAAAUACAGCUGUUUGGTT-3′). siGDF15#2 (sense: 5′- GACCUAUGAUGACUUGUUATT-3′, antisense: 5′- UAACAAGUCAUCAUAGGUCTT − 3′). The pcDNA3.1-GDF15 expression plasmid was synthesized by Genomeditech (Shanghai, China).

### ELISA

Human or mouse serum GDF15 levels were detected by ELISA kits (purchased from Suzhou Sizhengbai Biotechnology Co., LTD.). Then, 45 μl human serum was added to 180 μl sample diluent or 110 μl mouse serum to 110 μl sample diluent and incubated with the diluted biotin antibody and enzyme conjugate at 37 °C. After that, the colour-developing agent was added and incubated for 15 min away from light. The OD450 value was measured after the termination solution was added.

### Statistical analysis

SPSS 20 was used for analysis, and the chi-square (χ2) test was used for counting data. If the measurement data conformed to a normal distribution, the mean ± standard deviation was adopted; if they did not conform to a normal distribution, the median (front and back quartile) was adopted. For continuous data, the normality test was conducted first. If all groups met normality and the variance between the two groups was homogeneous, a t test was used for comparisons between groups. If the above conditions were not met, the nonparametric Mann‒Whitney U test was considered. The chi-square test was used for classification data and disordered classification data, and the nonparametric Mann‒Whitney U test was used for rank data. P < 0.05 was considered statistically significant.

## Results

### Upregulated of GDF15 in VTE patients

A total of 34 blood samples were collected in this study, including those from 17 patients in the VTE group (including 12 patients with PTE and 5 patients with PTE combined with DVT) and those from 17 patients in the control group. There were 18 males (52.9%) and 16 females (47.1%). The mean age was 62.1 ± 8.8 years in the VTE group and 60.1 ± 5.4 years in the control group. There was no significant difference in sex or age between the two groups. There were no significant differences in hypertension, diabetes, hyperlipidemia between the two groups. See Table 1 for details of patient characteristics.

**Fig. 1:**
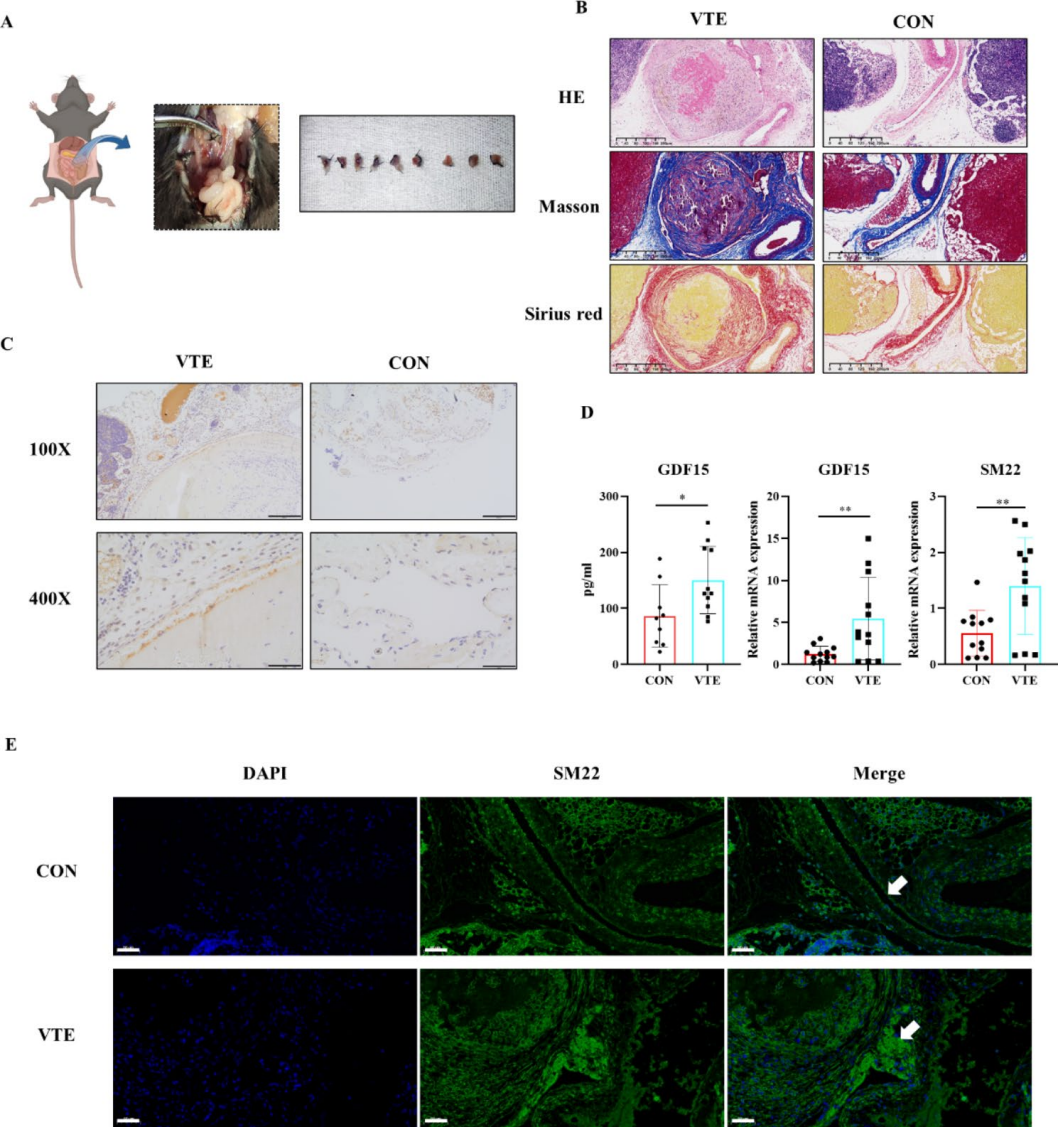
DVT modeling process and thrombus morphology in mice. (**A**) Mouse DVT model, ligation under the mouse inferior vena cava left renal vein. Thrombus specimens of mice 1 week after modeling; (**B**) Masson staining and Sirius red staining of control group and VTE group. The scale was 200 μm (100×). (**C**) (**D**) Expression of GDF15 in serum and tissue and EndMT marker in tissue. Detection of serum concentration of GDF15 by Elisa. Immunohistochemistry and RT-qPCR showed that GDF15 was highly expressed in the vascular endothelium in the VTE group, and the scale x10 was 200 μm (100×), 50 μm (400×). (**D**) (**E**) Fluorescence staining and RT-qPCR detection of mouse blood vessel sections showed increased expression of EndMT marker SM22, immunofluorescence blue was DAPI, green was SM22, the scale was 50 μm (400×). * p < 0.05

Combined with patient laboratory indicators and ELISA test results, statistical data were obtained. hsCRP and GDF15 levels in the VTE group were significantly higher than those in the control group: 2.8 (1.0,42.7) vs. 0.6 (0.4,0.9) mg/l, 568.72 (412.66,835.69) vs. 299.30 (271.00,440.14) pg/ml, p < 0.05 (Fig. 1). At the same time, homocysteine levels in the VTE group was significantly higher than that in the control group: 18.1 ± 6.7 vs. 11.6 ± 2.8 μmol/l. There were no significant differences in the distribution width of leukocytes, neutrophils, platelets, erythrocyte distribution width, thrombocytocrit, and CEA between the two groups. Detailed data are shown in Table 2.

**Table 1:** Baseline characteristics

Characteristics		VTE group(n = 17)	Control group(n = 17)
Age		62.1 ± 8.8	60.1 ± 5.4
Sex			
	male	8 (47.1%)	10 (58.8%)
	female	9 (52.9%)	7 (41.2%)
Past history			
	Hypertension	5 (29.4%)	3 (17.6%)
	Diabetes	5 (29.4%)	2 (11.8%)
	Hyperlipidemia	4 (23.5%)	4 (23.5%)
Thrombus type	PTE	12	
	PTE + DVT	5	

**Table 2:** Laboratory indicators

		VTE group	Control group
Leukocyte (10^9^/l)		6.7 ± 3.2	6.1 ± 1.1
Neutrophil (%)		62.0 ± 16.7	56.7 ± 10.2
Platelet(10^9^/l)		200.1 ± 60.2	184.6 ± 46.6
Erythrocyte distribution width (%)		13.1 ± 0.9	13.0 ± 0.6
Thrombocytocrit (%)		0.20 ± 0.05	0.20 ± 0.03
Homocysteine (μmol/l)		18.1 ± 6.7^*^	11.6 ± 2.8
hsCRP (mg/l)		2.8(1.0,42.7)^*^	0.6(0.4,0.9)
CEA (ng/ml)		2.56(1.51,17.7)	2.12(1.43,3.10)
GDF15 (pg/ml)		568.72(412.66,835.69)^*^	299.30(271.00,440.14)

*p<0.05

### Upregulated of GDF15 and SM22 in DVT mice model

Mice underwent DVT modelling, and mice in the control group underwent laparotomy and direct suturing. One week after modelling, mouse serum was collected according to the above steps, local tissues of the vena cava were sliced and stained, and tissue RNA was extracted for RT‒qPCR. After successful modelling in the VTE group, black thrombus tissue could be seen below the ligation line, and the local inferior vena cava was not easily compressed (Fig. 1A). HE staining of the thrombus sections showed that compared with the control group (CON group), the lumen of the inferior vena cava of mice in the VTE group was dilated, the lumen was full of thrombus tissue, there was partial cell infiltration in the thrombus and a large number of nonnucleated components. Masson staining and Sirius red staining indicated that there were more collagen fibre components in the thrombus (Fig. 1B). Serum GDF15 levels in the VTE group was significantly increased by ELISA (p < 0.05). Immunohistochemical staining of GDF15 showed that the expression of GDF15 in the vascular endothelium of the VTE group was higher than that of the control group (Fig. 1C). RT‒qPCR indicated that GDF15 mRNA expression was significantly upregulated and that the EndMT marker SM22 expression was increased in the local vascular tissue of the thrombus (Fig. 1D). Fluorescence staining of mouse vascular sections showed upregulated expression of SM22 in endothelial cells (Fig. 1E).

### Cell experiment

#### The role of EndMT and GDF15 induced by cytokine stimulation

**Fig. 2:**
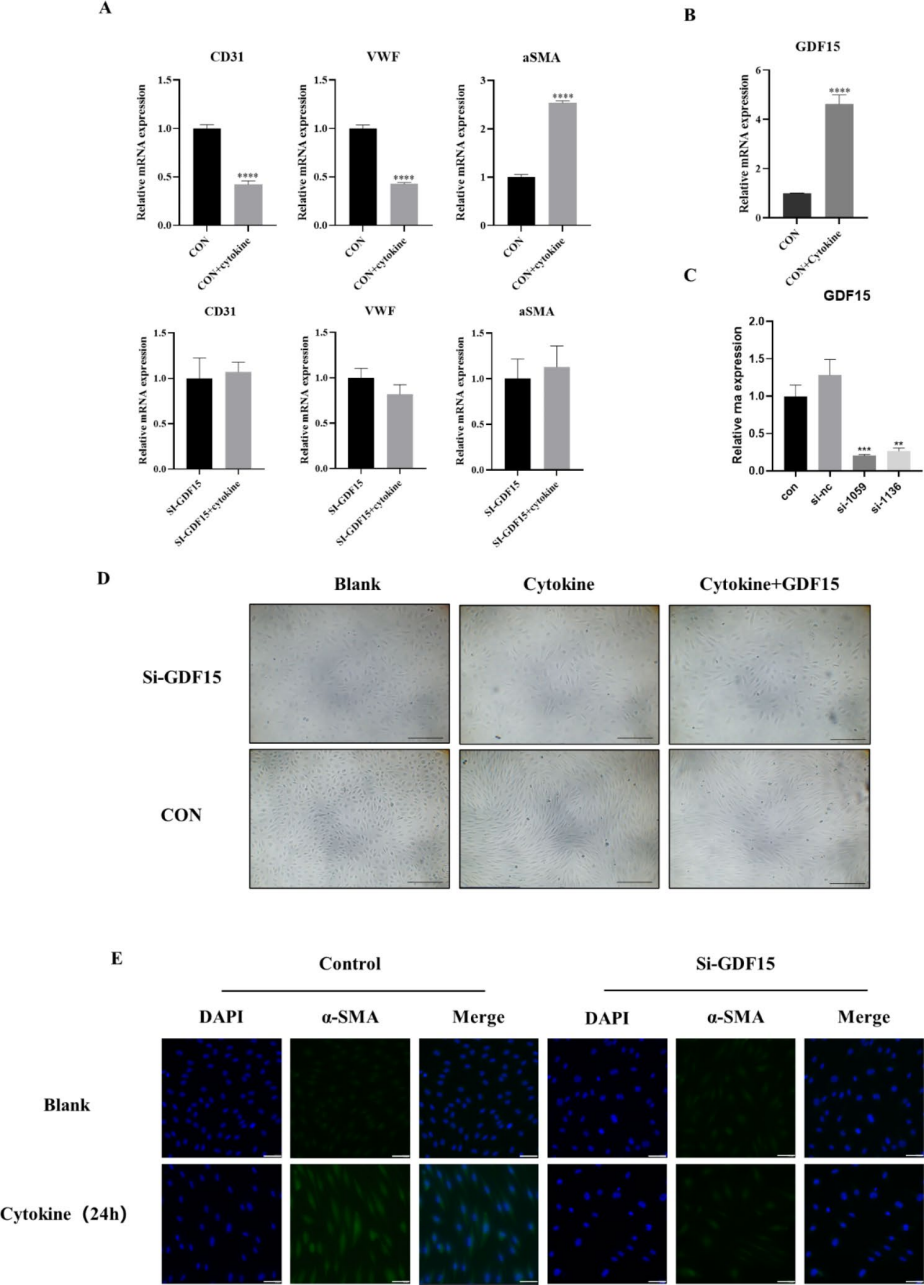
(**A**) RT-qPCR results indicated that endothelial marker (CD31, VWF) expression decreased, interstitial marker (α-SMA) expression was up-regulated, and GDF15 expression was significantly up-regulated after HUVECs were stimulated by the cytokine mixture. (**B**) RT-qPCR results suggested that the expression of GDF15 mRNA decreased after siRNA knockdown. (**C**) After GDF15 was knocked out, endothelial cells were stimulated by cytokines, and CD31, VWF and α-SMA were not significantly changed, * compared with CON group. (**D**) In addition to knockout, cytokine treatment showed significant relief in promoting endothelial interstitial transformation and reduced morphological changes in endothelial cells, the scale was 500 μm, (40×); (**E**) Immunofluorescence assay was performed after 24 h stimulation with cytokine mixture, and the expression of EndMT-induced α-SMA was significantly reduced by knockout of GDF15, the scale is 50 μm (400×). Significant post hoc effects were revealed by the Bonferroni post hoc test. *p < 0.05, **p < 0.01, ***p < 0.001, ****p < 0.0001

In this study, cytokines were jointly stimulated by a TGF-β1 + TNF-α + IL-1β mixture (mixed concentrations were 5 ng/ml, 5 ng/ml, and 0.1 ng/ml, and the mixture was expressed by subsequent cytokines). After 24 h of cytokine stimulation, the morphology of endothelial cells changed from pavers to spindles under a light microscope (Fig. 2D). Total mRNA was extracted from cells according to the preceding steps, and RT‒qPCR detection indicated that endothelial marker (CD31, VWF) expression decreased, while interstitial marker (α-SMA) expression was upregulated (Fig. 2A). In addition, GDF15 mRNA expression was significantly upregulated after cytokine stimulation (Fig. 2B). The expression of GDF15 decreased significantly after the instantaneous knockout of GDF15 using siRNA (Fig. 2C), indicating successful knockout. In addition to knockout, cytokine treatment showed significant relief in promoting endothelial interstitial transformation and reduced morphological changes in endothelial cells (Fig. 2D), and immunofluorescence showed that GDF15 inhibited cytokine-induced upregulation of α-SMA (Fig. 2E).

**Fig. 3:**
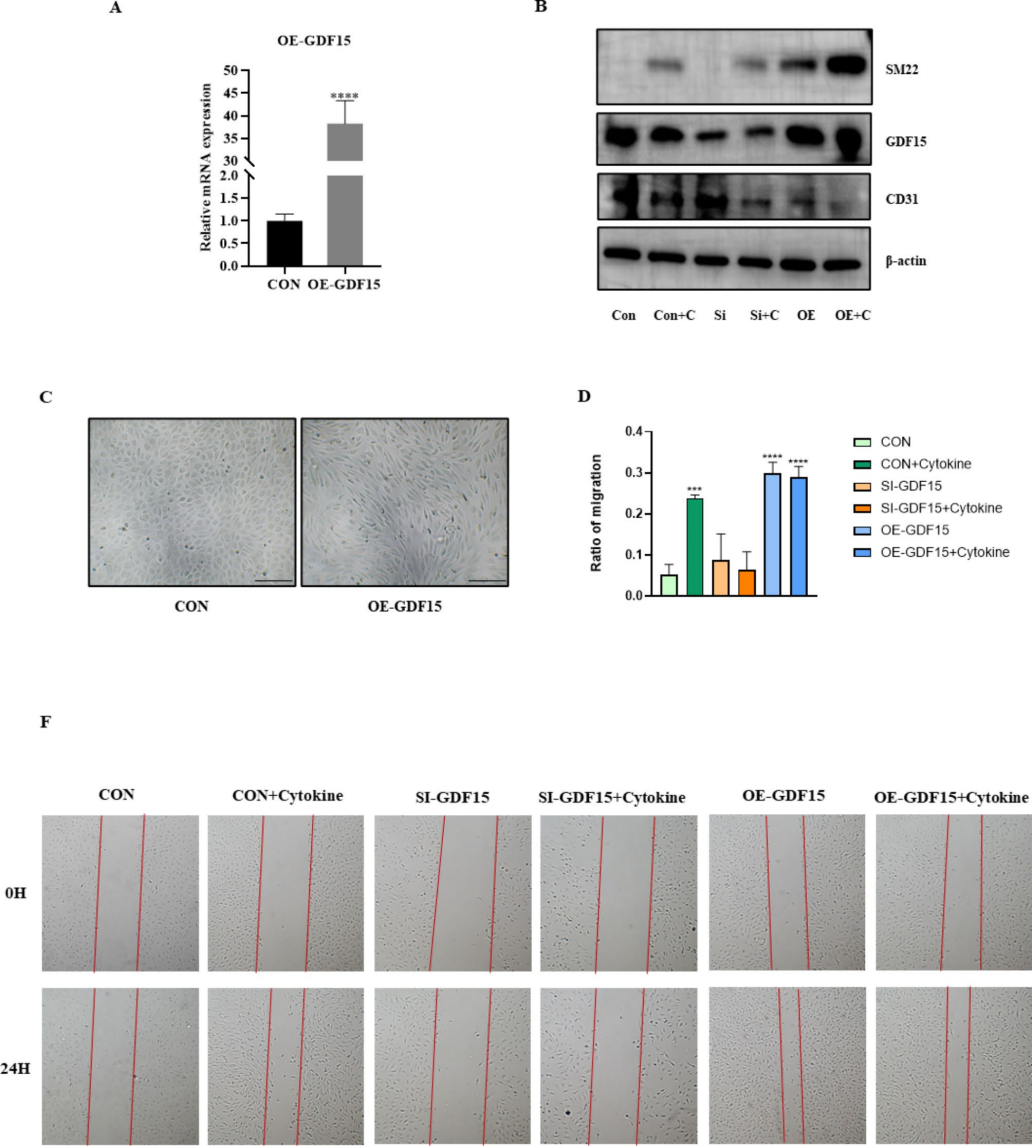
Cell morphology, mRNA expression, WB results. (**A**) After endothelial cells transfected with overexpression of GDF15 plasmid, the mRNA expression of GDF15 was significantly up-regulated by RT-qCPR. (**B**) Western blot assay was used to detect GDF15 protein expression changes in GDF15 knockout cells and overexpressed cells, as well as endothelial and interstital marker changes after cytokine treatment, in which CON was the control group, CON + C was the control group + cytokine, Si was GDF15 knockout, OE was overexpressed GDF15. (**C**) After transfected with GDF15 plasmid and continued culture for 48 H, messtitial cell characteristics appeared in the morphology of endothelial cells, with a scale of 500 μm (40×). Cell scratch experiments (**D**, **E**) To reduce the influence of cell activity changes and proliferation on the scratch experiment results, we starved the cells for 24 H in advance, and used 1% serum during the experiment. The crawling ability of endothelial cells was enhanced after cytokine stimulation or transfection of the overexpressed plasmid, while the crawling ability of endothelial cells induced by cytokine stimulation was decreased by instantaneous knockout of GDF15, scale 500 μm (40×). * in Figure D represents comparison with CON group, significant post hoc effects were revealed by the Bonferroni post hoc test. ***p < 0.001, ****P < 0.0001

According to the preceding steps, the vector plasmid GDF15 pcDNA3.1 (+) was constructed, and the expression of GDF15 in endothelial cells was significantly upregulated after transfection (Fig. 3A). Morphology showed spindle changes in HUVECs 48 h after transfection with the overexpressed GDF15 plasmid (Fig. 3C). Protein electrophoresis results showed that GDF15 protein expression was decreased after transfection with GDF15-siRNA, CD31 expression was decreased after transfection with the overexpressed plasmid, and SM22 expression was significantly upregulated (Fig. 3B). Data of other EndMT-makers were shown in Supplement 2 Fig. 1. To further explore the functional changes in endothelial cells after mixed factor stimulation and overexpression of GDF15, scratch experiments were performed, and the results showed that the crawling ability of endothelial cells was enhanced after cytokine stimulation or transfection of the overexpressed plasmid, while the crawling ability of endothelial cells induced by cytokine stimulation was decreased by instantaneous knockout of GDF15 (Fig. 3D, E). Data of cell apoptosis were included in Supplement 2 Fig. 2.

#### The effect of EndMT induced by a cytokine mixture on coagulation and fibrinolysis as well as the function and possible mechanism of GDF15

**Fig. 4:**
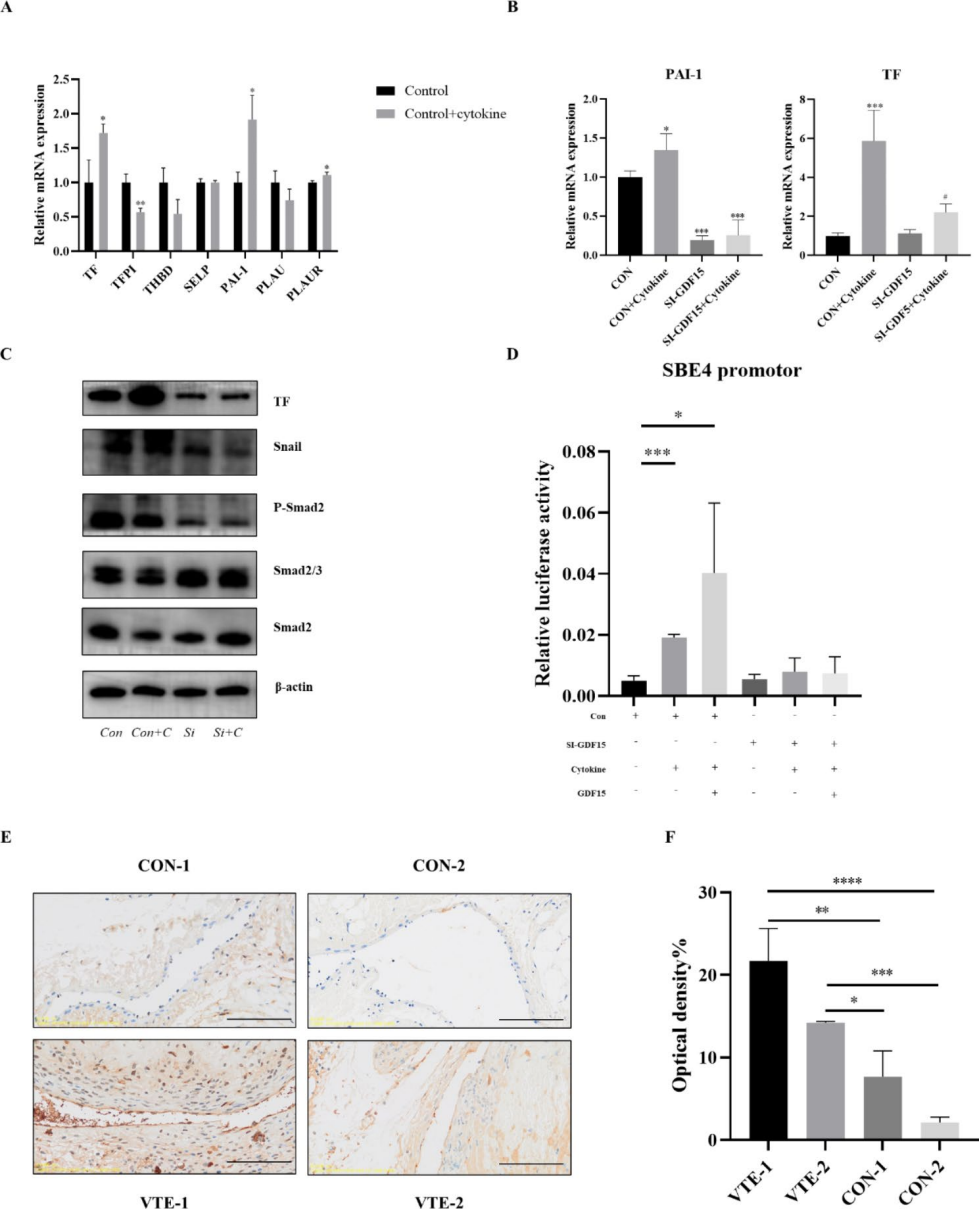
Cell mRNA expression, WB and double luciflucidase assay. (**A**) After stimulating endothelial cells with TGF-β1 + TNF-α + IL-1β for 24 h, RT-qPCR was used to detect mRNA levels of related proteins. TF and PAI-1 expressions were up-regulated, while TFPI and PLAU expression levels were down. (**B**) After 24 h stimulation of endothelial cells by cytokine mixture, TF and PAI-1 expression decreased significantly after GDF15 knockout, *compared with CON group, ^#^ compared with CON + cytokine group. (**C**) Western-blot results, where CON is the control group and CON + C is the control + cytokine. After cytokine stimulation, Smad pathway was activated in endothelial cells. Knocking down GDF15 inhibited the phosphorylation of Smad2 and the expression of the transcription factor snail, while TF expression decreased significantly. (**D**) Dual luciferase reporter gene, SBE4 transcription level is up-regulated after EndMT, and knocking down GDF15 can significantly inhibit this effect. (**E**, **F**) Results of p-smad2 immunohistochemical staining of mouse inferior vena cava tissue, the scale was 200 μm (100×). Average optical density value of the positive signal of immunohistochemistry analyzed by Image J. Significant post hoc effects were revealed by the Bonferroni post hoc test. *P < 0.05, **P < 0.01, ***P < 0.001, ****P < 0.0001

Combined with the above results, we found that GDF15 plays a role in the process of inflammatory cytokines stimulating endothelial cells to produce EndMT. Next, we further explored the changes in the coagulation and fibrinolytic system of endothelial cells after EndMT, as well as the role and possible mechanism of GDF15 in this process. After the mixture of cytokines stimulated endothelial cells for 24 h, endothelial cells showed procoagulant enhancement (e.g., tissue factor (TF) increased, tissue factor pathway inhibitor (TFPI) decreased, antifibrinolytic enhancement (e.g., plasminogen activator inhibitor-1(PAI-1) upregulation, and urokinase-type plasminogen activator (uPA/PLAU) decreased) as shown in Fig. 4A. Data of thrombin was shown in supplement 2 Fig. 3. After GDF15 knockout, the expression of PAI-1 and TF was significantly inhibited (Fig. 4B). Western blot analysis showed that transfection of siRNA significantly reduced the phosphorylation level of Smad2 and the expression of the transcription factor Snail (Fig. 4C). The double luciferase reporter gene assay suggested that GDF15 knockout reduced the transcription level of the SBE4 promoter (Fig. 4D). Immunohistochemical staining of p-smad2 (Abmart, T55859) on the inferior vena cava tissues indicated that p-smad2 expression was elevated in the vascular tissues of the VTE group (Fig. 4E F). In conclusion, knockout of GDF15 can reduce EndMT in endothelial cells by inhibiting Smad2 phosphorylation to alleviate cytokines, thereby improving the coagulation and fibrinolytic function of endothelial cells.

#### Effect of inhibition of GDF15 expression on venous thrombosis in mice

**Fig. 5:**
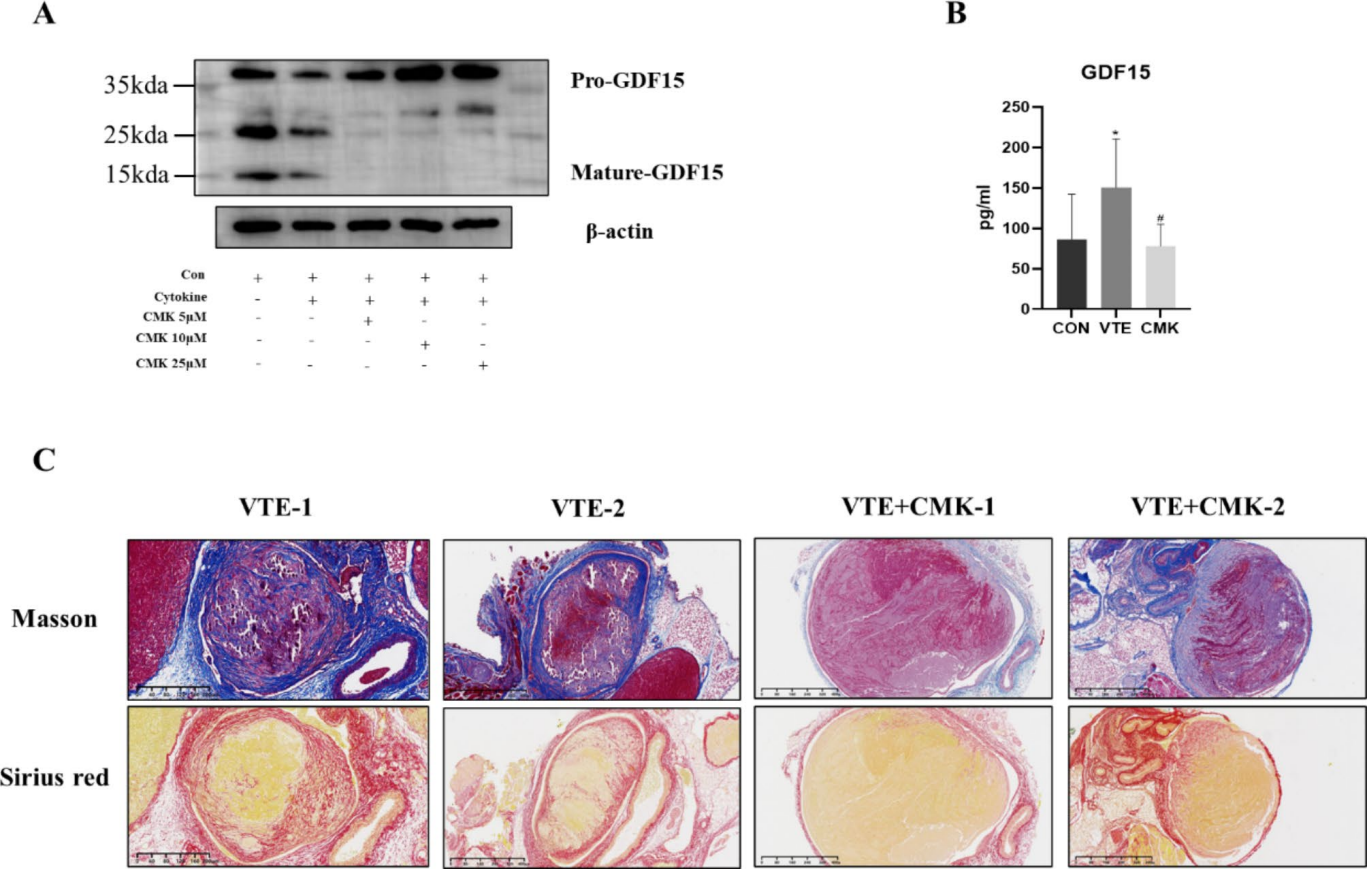
WB results, mouse serum GDF15 and blood clot section staining after drug treatment. (**A**) WB results showed that on the basis of cytokine stimulation, CMK significantly inhibited the maturation of GDF15. (**B**) Elisa was used to detect the serum GDF15 level of mice. There was no significant increase of GDF15 in the CMK inhibitor group after modeling, and the scale was 200 μm (100×). *, ^#^ p < 0.05, * is compared with CON group, and # is compared with VTE group. (**C**) Masson and Sirius red staining showed that the content of thrombe fibers in the CMK treated group of mice was reduced (Masson fibers were dyed blue, Sirius red fibre dyed red), the scale was 200 μm (100×)

The precursor of GDF15 needs to be further processed into a mature protein in the cell before it can be secreted into the extracellular space. Decanoyl-RVKR-CMK (later abbreviated as CMK) is a subtilis protease/Kex2p-like proprotein invertase inhibitor [26]. Previous studies have reported that this inhibitor can significantly inhibit the transformation of GDF15 from the precursor to the mature body, thereby reducing its exocrine level. Therefore, CMK (5 μM, 10 μM, 25 μM) was added based on cytokine treatment according to the concentration gradient, and WB results showed that CMK significantly inhibited the maturation of GDF15 (Fig. 5A). Subsequently, we used this inhibitor in a mouse model of deep vein thrombosis. Normal saline (0.9%) was used to dissolve the drug, and the drug was injected through the tail vein on Day 0 and Day 3 after modelling, while the control group was injected with the same amount of normal saline. Serum ELISA of mice showed that GDF15 was significantly decreased in the CMK treatment group compared with the normal modelling group (Fig. 5B), and the fibre components in the thrombus of mice were decreased in the CMK treatment group compared with the control group after section staining (Fig. 5C).

**Fig. 6:**
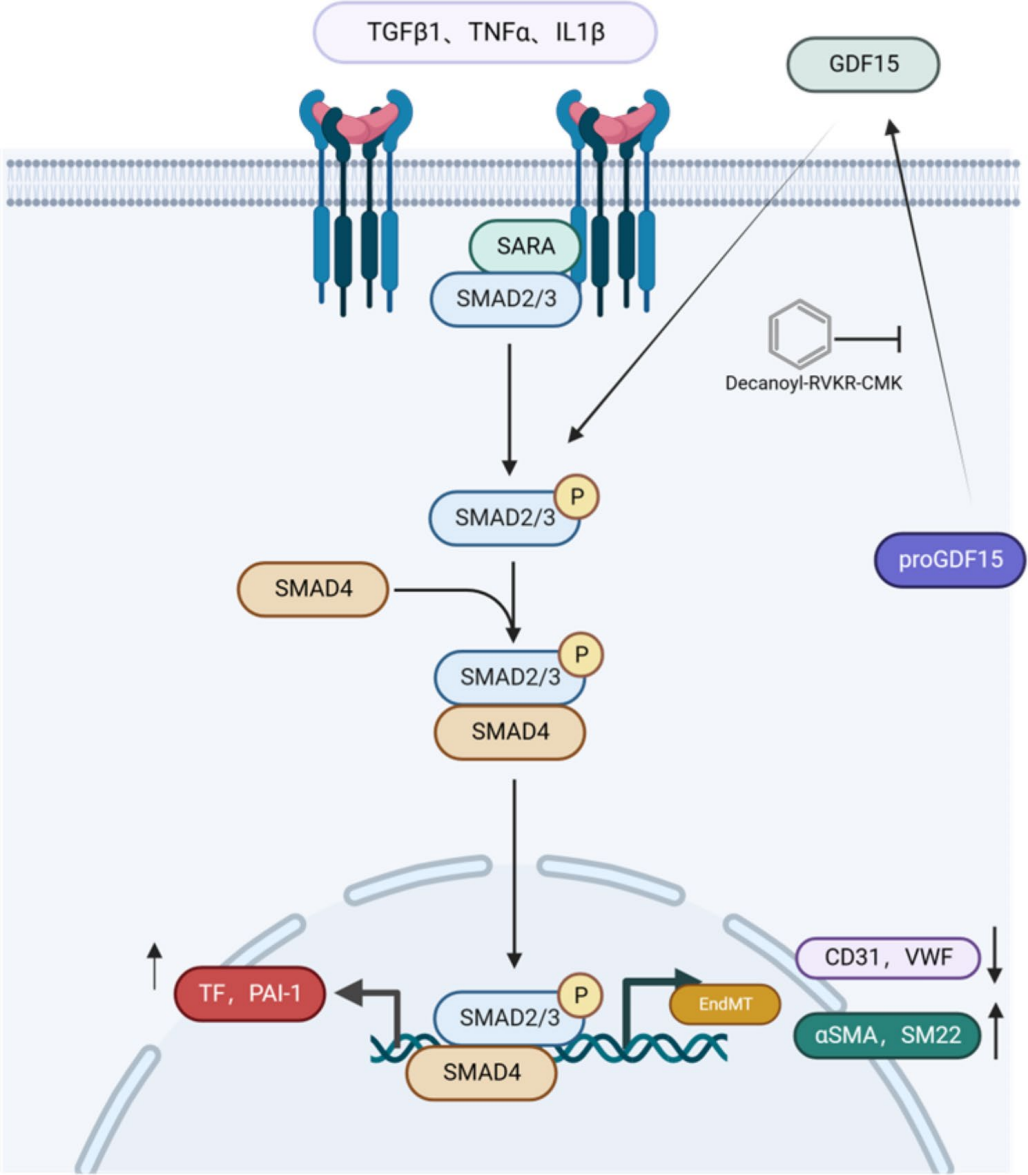
The possible mechanism of GDF15 in VTE. GDF15 plays an important role in the cytokine-induced EndMT process of endothelial cells. Inhibition of GDF15 expression can inhibit the EndMT process, further inhibit the coagulation and improve the fibrinolysis function of endothelial cells, and thus reduce the local fiber deposition of thrombosis

## Discussion

At present, immune thrombosis has become a popular topic in the field of VTE. An increasing number of people have realized that the occurrence of VTE is related to immunity and inflammation, which cannot be ignored. There is a significantly increased risk of venous thromboembolism in some conditions associated with inflammation, particularly surgery, obesity, sepsis, cancer, inflammatory bowel disease (IBD), and COVID-19 [27]. In this study, we found that the inflammatory factor GDF15 can activate EndMT through the Smad2/psmad2/snail pathway to promote thrombosis, while inhibition of GDF15 secretion can reduce fibre deposition in thrombi in mouse DVT models. A diagram of the possible mechanisms is shown in Fig. 6.

In this study, hsCRP in the VTE group was significantly higher than that in the control group. High-sensitivity C-reactive protein (hsCRP), a member of the highly conserved pentosan superfamily of proteins, is often used as a marker of infection and inflammation in clinical practice. Currently, there is increasing evidence that hsCRP is not only a marker of inflammation but also that the unstable C-reactive protein isomer has proinflammatory and prothrombotic properties [27]. hsCRP has a direct effect of amplifying thrombus inflammation in ischaemia/reperfusion injury and transplant rejection, and inhibition of the CRP subtype (pCRP) can have anti-inflammatory effects [27].

In addition, consistent with the clinical data in Part I, serum levels of GDF15 in the mouse model of deep vein thrombosis were significantly higher than those in the control group. mRNA extracted from local vascular tissues and detected by RT‒qPCR also showed high expression of GDF15, accompanied by high expression of the interstitial marker SM22, indicating the local occurrence of EndMT in the vascular wall. Endothelial cells have been found to acquire mesenchymal phenotypes through a process called EndMT. EndMT first occurs in developing embryos and can be triggered after birth under certain pathological conditions. In this process, ECs dedifferentiate into mesenchymal stem cell-like cells (MSCs), which subsequently produce cell types belonging to the mesoderm lineage. Because EndMT contributes to a variety of diseases, pharmacological regulation of EndMT signaling pathways may be an effective therapeutic approach [28].

It has been reported that EndMT can be induced by inflammatory factors, including TNFα, IL-1β, TGFβ1, and TGFβ2. Inflammation-induced EndMT is primarily controlled by two signalling pathways: transforming growth factor β (TGFβ) and non-TGFβ. Bochenek et al. found that endothelial TGFβ signaling pathways and EndMT are important drivers of CTEPH. The thrombus ablation in established VTE models in platelet-specific TGFβ1-deficient mice and endothelium-specific TGFβRII-deficient mice was significantly accelerated in TGFβ1-deficient mice, while endothelium-specific TGFβRII deficiency significantly delayed thrombus resolution. Thrombi from mice with endothelial-specific TGFβRII deletion showed the characteristics of EndMT, such as increased fibrosis, increased collagen expression, and CD31-positive cells coexpressing FSP-1 or SMA. Immunohistochemical analysis of CTEPH patient samples showed that the TGFβ signaling pathway was overactivated, which was related to high circulating levels and overexpression of TGFβ [29]. Other pathways that regulate EndMT include Akt/NF-κB [30], Notch1 [31], MAPK [32], PI3K [33], and sp1 [34].

Most literature used single-factor stimulation, some scholars used a mixture of two cytokines, and some literature used the combined stimulation of three cytokines in PAECs (pulmonary artery endothelial cells) [35] and HIMECs (human intestinal microvascular endothelial cells) [34]. Different single factors or mixtures of cytokines and endothelial cell types have different responses to EndMT. In this study, TGFβ1, IL1β, and TNFα were combined to stimulate HUVECs. RT‒qPCR and Western blot analysis confirmed the changes in EndMT-related markers, including the decrease in the levels of CD31 and VWF and the increase in α-SMA and SM22 expression. At the same time, endothelial cells had changes in coagulation and fibrinolytic functions, manifested as coagulation promotion and fibrinolytic inhibition (TF and PAI-1 expression increased), which might be related to the potential mechanism of EndMT’s promoting effect in the development of thrombi. Knockout of GDF15 mitigates the increase of thrombin activity attracted by cytokine stimulation. In addition, we found that GDF15 plays a key role in this process. Both knockdown and overexpression of GDF15 affect the functional transformation of endothelial cells possibly by the Smad2/psmad2/snail pathway.

GDF15 is one of the stress response members of the TGF-β cytokine superfamily; however, the sequence conservation rate with other superfamily members is 15–29% [16]. Although it is named growth differentiation Factor 15 (GDF15), recent data suggest that it is not a growth differentiation factor and is more likely a glia-derived neurotrophic factor due to its high affinity for glia-derived neurotrophic factor (GDNF) receptor-alpha-like (GFRAL) [36]. Although it has been reported that GDF15 can activate the downstream pathway through TGF-βR in some specific cells (e.g., cardiomyocytes, colon cancer cells, etc.), it is not clear whether GDF15 can act through TGF-β family receptors because of the purification of recombinant proteins [37]. Therefore, in this paper, the role of GDF15 in endothelial cells was studied mainly through overexpression and transient knockout. In addition, decanoyl-RVKR-CMK was used in this study to inhibit the maturation of GDF15 protein, reduce its exocrine effect and reduce thrombus fibre deposition in a mouse thrombotic model. GDF15 has various forms in cells, including pro-GDF15 monomer (~ 40 kDa), pro-GDF15 dimer (~ 80 kDa), and mature dimer (~ 30 kDa). Currently, most studies focus on the mature form of GDF15, and little attention has been given to its precursors. K-W Min et al. found that the precursor of GDF15 may inhibit the smad pathway in the nucleus by inhibiting the binding of the smad complex to the downstream promoter [38]. In this study, GDF15 played a role in promoting EndMT in endothelial cells. The specific function and mechanism of mature GDF15 and its precursors need to be further explored. In conclusion, GDF15 plays an important role in the cytokine-induced EndMT process of endothelial cells. Inhibition of mature GDF15 expression can inhibit the EndMT process, further improving the coagulation and fibrinolytic function of endothelial cells and thus reducing the local fibre deposition of thrombi.

The limitation of this paper is that only the serum level of GDF15 at the onset of VTE was studied. Long-term follow-up and monitoring of GDF15 levels can be arranged in the future to evaluate its relationship with the subsequent remission of VTE and chronic lesions. Second, this paper only studied the effect of GDF15 on EndMT in HUVEC cell lines but did not study whether there were differences in the effects of GDF15 on other endothelial cells. Further studies on more types of endothelial cells, such as pulmonary artery endothelial cells, are needed in the future.

## Conclusions

GDF15 positively promotes EndMT through activation of the Smad2/psmad2/snail pathway, and inhibition of GDF15 expression can alleviate the EndMT process, further improving the coagulation and fibrinolytic function of endothelial cells and thus reducing the local fibre deposition of venous thrombi.

### Electronic supplementary material

Below is the link to the electronic supplementary material.

Supplementary Material 1

Supplementary Material 2

## Data Availability

The data used and/or analyzed during the current study are available from the corresponding author upon reasonable request.

## Abbreviations

BCA: Bicinchoninic acid
DVT: Deep vein thrombosis
ECM: Endothelial cell culture-medium
Elisa: Enzyme linked immunosorbent assa
EndMT: Endothlial-mesenchymal transition
GDF15: Growth differentiation factor15
hsCRP: High-sensitivity c-reactive protein
HUVECs: Human umbilical vein endothelial cells
IL-1β: Interleukin-1β
PAI-1: Plasminogen activator inhibitor-1
RT-qPCR: Quantitative real-time PCR
SM22: Smooth muscle protein 22-α
TGFβ: Transforming growth factor-β
TNFα: Tumor necrosis factor-α
VTE: Venous thrombus embolism
