# Supplementary material for: GDF15 affects venous thrombosis by promoting EndMT through smad2/p-smad2 pathway
Source: Thromb J. 2023 Sep 18;21:98. doi: 10.1186/s12959-023-00547-7 (PMC10506185; doi:10.1186/s12959-023-00547-7)
Supplement: Supplementary file 2 — Supplementary Material 2 [file 12959_2023_547_MOESM2_ESM.pdf]

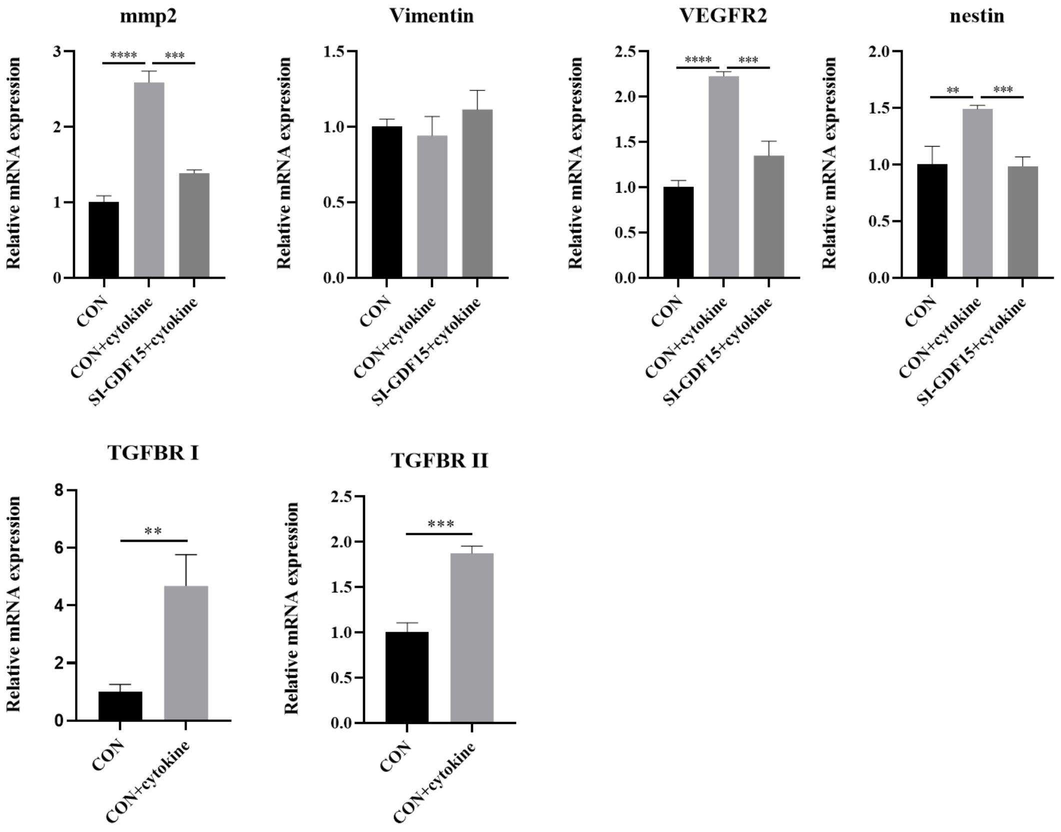

Fig.1 Relative mRNA expression changes of some other EndMT markers such as VEGFR2, TGFbetaR I, TGFbetaR II, nestin, mmp2 and Vimentin. Significant post hoc effects were revealed by the Bonferroni post hoc test. **P < 0.01, ***P < 0.001, ****P < 0.0001.

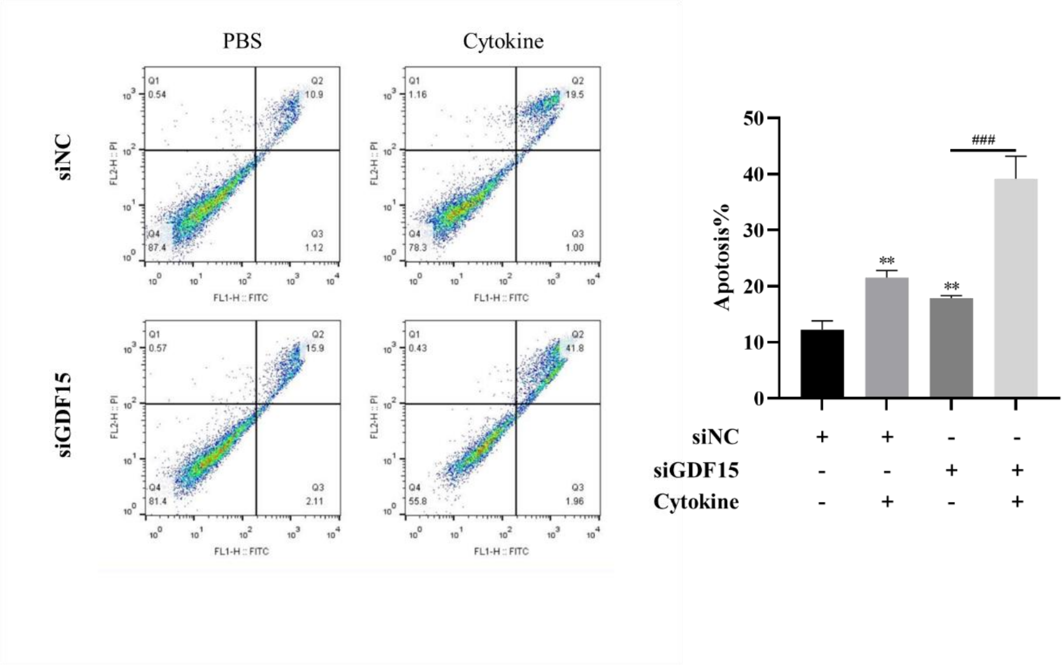

Fig.2 Cell apoptosis detected by flow cytometry. GDF15 knockout alleviate the apoptosis of endothelial cells induced by cytokines. Significant post hoc effects were revealed by the Bonferroni post hoc test, *compared with siNC group, # compared with siGDF15 group. **P < 0.01, ***P < 0.001.

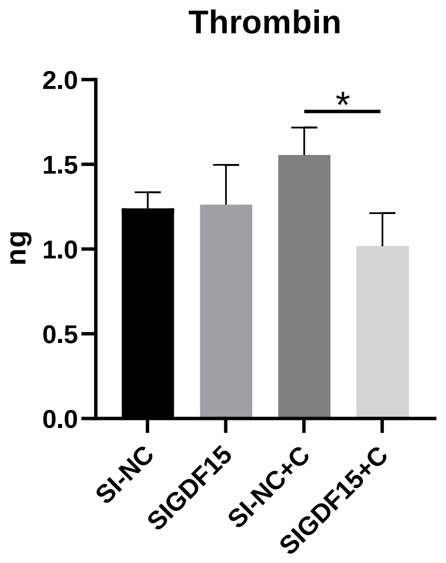

Fig.3 Thrombin activity was detected by thrombin activity fluorometric assay kit (Sigma, MAK242). In the presence of cytokines, thrombin activity was lower in the GDF15 knockout group. Cytokines can promote the increase of thrombin activity, although there is no statistical difference. *P < 0.05.
